# Analysis of the virus propagation profile of 14 dengue virus isolates in *Aedes albopictus* C6/36 cells

**DOI:** 10.1186/s13104-020-05325-6

**Published:** 2020-10-12

**Authors:** Atitaya Hitakarun, Suwipa Ramphan, Nitwara Wikan, Duncan R. Smith

**Affiliations:** Molecular Pathology Laboratory, Institute of Molecular Biosciences, Mahidol University, Salaya Campus, 25/25 Phuttamonthon Sai 4, Salaya, Nakhon Pathom, 73170 Thailand

**Keywords:** Dengue virus, Genetic diversity, Virus production profile

## Abstract

**Objective:**

The mosquito transmitted RNA virus dengue virus (DENV) shows significant variation as a consequence of the lack of proofreading activity of the RNA-dependent RNA polymerase that synthesizes new virus genomes. How this variation affects DENV replication, and how this in turn impacts drug development remains largely unknown. Given the technical limitations in working with large numbers of isolates few studies have sought to investigate this area. This study used a panel of 14 DENV isolates of different serotypes and origins to determine how much virus replication in *Aedes albopictus* C6/36 cells was affected by DENV variability.

**Results:**

The results showed that there was considerable variation, with peak titers ranging from 6Log10 to 8Log10, and maximum titer being reached from day 3 to day 9 post infection. While strains from DENV 1 and 4 serotypes showed considerable uniformity, DENV 2 and 3 strains showed much greater variation. Overall, these results show that serotype specific strain variation can have a significant impact on DENV replication, suggesting that studies either investigating DENV pathogenesis or developing drug therapeutics should consider the contribution of DENV variability.

## Introduction

Dengue virus (DENV) is an enveloped virus and composed of a single-stranded RNA genome encoding for three structural proteins (capsid (C), pre-membrane (prM), and envelope (E)) and seven non-structural (NS) proteins (NS1, NS2A, NS2B, NS3, NS4A, NS4B, and NS5) [1]. The NS5 protein is the largest and most conserved protein and encodes a methyltransferase in the N-terminal domain and a RNA-dependent RNA polymerase (RdRP) in the C-terminal domain, with the domains being separated by a short flexible linker [2]. As with other RNA viruses, the DENV RdRP lacks proofreading activities, and as such, DENV has considerable variation, with each serotype consisting of multiple lineages each of which consists of many different strains [3].

In a previous study we evaluated the replication kinetics of four laboratory adapted DENVs in *Aedes (Ae.) albopictus* C6/36 cells, investigating one strain for each serotype [4]. The results of that study showed that there was very little variation in virus production kinetics over an intermediate time period (up to 15 days post infection), and that the titers of the four DENVs differed by less than 1Log_10_. Given that we have collected a relatively large panel of low passage DENVs of different origins [5, 6], this study sought to determine the replication kinetics of these viruses to determine whether a relatively uniform virus production as seen with four laboratory adapted viruses [4] was consistent in a larger panel of viruses. For comparison, one high passage isolate was also included.

## Main text

### Materials and methods

#### Cell lines and cell culture conditions

C6/36 cells (ATCC^®^CRL-1660™) were cultured at 28 °C in minimum essential medium (MEM, Thermo Fisher Scientific, Waltham, MA). MEM was supplemented with 10% fetal bovine serum (FBS, Thermo Fisher Scientific, Waltham, MA) and 100 units/ml of penicillin/streptomycin solution (Pen/Strep, Merck KGaA, Darmstadt, Germany) and ambient CO_2_. LLC-MK_2_ cells (ATCC^®^CCL-7™) were cultured at 37 °C with 5% CO_2_ in Dulbecco’s modified Eagle’s medium (DMEM, Thermo Fisher Scientific, Waltham, MA). DMEM was supplemented with 5% FBS and 100 units/ml of Pen/Strep.

#### Virus propagation

The DENVs used in this study have been partially sequenced for virus identification as previously described [5]. The representative viruses include one high passage isolate of DENV 1 Hawaii strain (DENV 1Hawaii) and thirteen low passage isolates. The low passage isolates had all been passaged less than 5 times in C6/36 cells, and included four low passage isolates originally from dengue fever (DF) patients, namely DENV 1 strain SS12/61 (DENV 1DF), DENV 2 strain SS12/62 (DENV 2DF), DENV 3 strain SS12/64 (DENV 3DF), and DENV 4 strain SS12/66 (DENV 4DF), four low passage isolates originally from dengue haemorrhagic fever (DHF) patients, namely DENV 1 strain SS12/60 (DENV 1DHF), DENV 2 strain SS12/63 (DENV 2DHF), DENV 3 strain SS12/65 (DENV 3DHF), and DENV 4 strain SS12/67 (DENV 4DHF), and five low passage isolates originally from undifferentiated fever patients, namely DENV 1 strain SS11/1666 (DENV 1/1666), DENV 3 strain SS15/1113 (DENV 3/1113), DENV 4 strain SS11/1373 (DENV 4/1373), DENV 4 strain SS14/146 (DENV 4/146), and DENV 4 strain SS14/163 (DENV 4/163). All DENV isolates were propagated in C6/36 cells as described previously [5] and were stored at −80 °C until required. Provenance of all viruses can be found in Additional file 1: Table S1.

#### Virus production profiles

C6/36 cells were seeded into 25 cm^2^ tissue culture flasks (Corning, NY, USA) at a density that allowed 90–95% confluency to be reached within 48 h in an incubating oven at 28 °C without CO_2_. The cells were infected with DENV at a multiplicity of infection (MOI) of 0.1 in MEM (with no FBS or antibiotic) for 2 h with constant agitation. The infected cells were washed with 1X phosphate buffered saline (PBS). Extracellular viruses were inactivated using acid glycine (pH3) for 1 min [7] and the cells were immediately washed with 1X PBS and complete MEM. Cells were maintained in fresh MEM with 10% FBS but without antibiotics at 28 °C. The culture media were collected at different time points from early-term (0, 3, and 6-hours post-infection; h.p.i.) to intermediate-term (every day for 15 days) and stored at −80 °C until use. After sample collection, an equal volume of fresh complete MEM was added to substitute the media removed. Each virus was analyzed independently in triplicate. The titers of all collected samples were assayed in duplicate standard by plaque assay on LLC-MK_2_ cells as described previously [5], and plaques were counted manually. The viral replication curves were plotted as mean ± the standard deviation (SD) using GraphPad Prism version 7.0a (GraphPad Software Inc., San Diego, CA). Raw data of viral titers is provided in Additional file 2. Statistical analysis was undertaken with a one-way ANOVA (non-parametric test with Kruskal–Wallis test) for DENV 1, DENV 3, DENV 4, DENV DF, DENV DHF, and DENV undifferentiated fever samples, while a *t* test (non-parametric test with Mann–Whitney test) was used for DENV 2 due to the limited number of strains. All statistical analyses were undertaken using GraphPad Prism version 7.0a (GraphPad Software Inc., San Diego, CA).

#### Phylogenetic tree analysis

The identity of all 14 DENV isolates was confirmed by RT-PCR amplification of a portion of the genome corresponding to the capsid and pre-membrane sequences using primers D1 and D2 [8] exactly as described elsewhere previously [5]. The nucleotide sequence of each strain was assembled from both the forward and reverse stands using SeqMan Pro version 8.1.3 (DNASTAR, Inc., Madison, WI) before being converted into the corresponding amino acid sequence using A Plasmid Editor program (ApE, version 2.0; http://www.softsea.com/review/ApE-A-Plasmid-Editor.html). All 14 amino acid sequences were aligned and the pairwise genetic distances among strains determined using the Molecular Evolutionary Genetics Analysis software (MEGA, version 10.1.6; http://www.megasoftware.net/). The phylogenetic tree was constructed using the maximum likelihood method through MEGA version 10.1.6 programs with 1000 bootstrap replications.

### Results and discussion

#### Virus production profiles

**Fig. 1:**
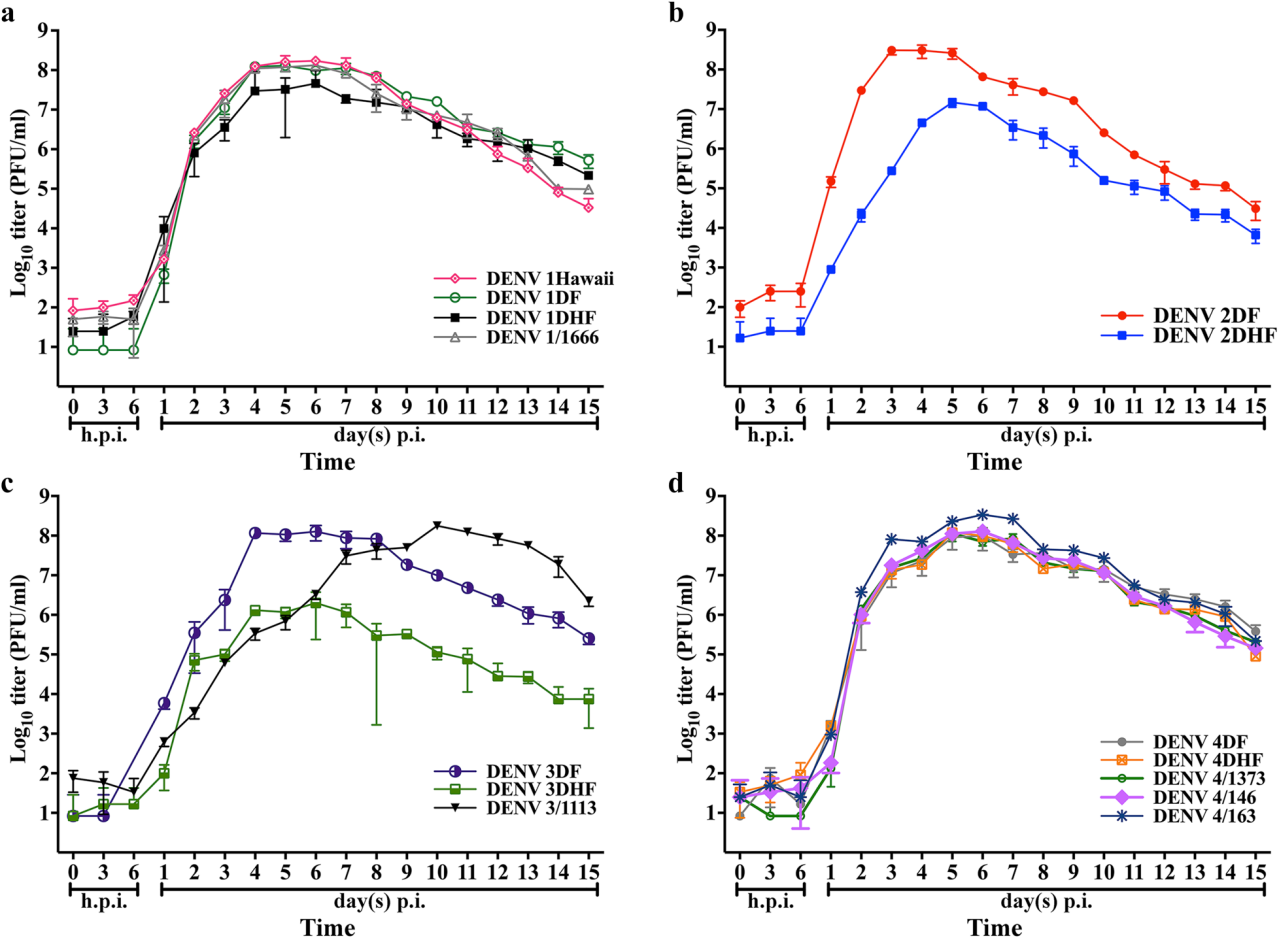
Growth kinetic production profiles of DENV classified by serotype. C6/36 cells were infected with different isolates of all four dengue serotypes (DENV 1 - DENV 4) individually at a MOI of 0.1 and the levels of infectious viruses at different time points were assayed by duplicate standard plaque assays on LLC-MK_2_ cells. The virus production profiles were classified by serotype. The serotypes of DENV included (**a**) serotype 1 (**b**) serotype 2 (**c**) serotype 3, and (**d**) serotype 4. The experiment was undertaken independently in triplicate and error bars show mean ± SD. A full statistical analysis of the data is provided in Additional file 2

The issue of DENV variation, and how this affects virus interactions with both insect and mammalian host cells remains poorly investigated. In part this is a simple matter of practicality, as with four DENV serotypes, the number of isolates that need to be investigated to provide a comprehensive picture can quickly become technically prohibitive. However, the matter is important, as we have previously shown that strain variation can affect the type of proteins being differentially regulated in LLC-MK_2_ cells upon DENV infection [6], as well as the effectiveness of a particular drug treatment in reducing DENV infection [5]. To look at whether strain variation affects virus replication kinetics we investigated the early (0 to 6 h.p.i.) and intermediate (up to 15 days) replication profile of 14 DENV strains isolated from patients with different disease severities (undifferentiated fever, DF, DHF), including 1 high passage isolate and 13 low passage isolates. Representative strains from all four DENV serotypes were examined. The identity of all viruses used was confirmed by partial sequencing as described previously [5] using primers that amplify a portion of the genome corresponding to the capsid and pre-membrane sequences [8], and a phylogenetic tree based on the sequences is given in Additional file 1: Figure S1. To determine the replication profile in C6/36 cells, C6/36 cells were individually infected at an MOI of 0.1 followed by treatment with glycine pH 3.0 to remove uninternalized extracellular viruses [7]. Figure 1 shows a comparison of growth production profiles of the isolates by serotype (irrespective of strain origin). All of the strains showed a maximum titer of 7Log_10_ to 8Log_10_ (Fig. 1a–d), with the exception of DENV 3DHF which showed a maximum titer of only 6Log_10_ (Fig. 1c). This is in very good agreement with our previous observations seen with four high passage, laboratory adapted viruses with one strain representative of each serotype [4]. For most viruses the highest titer was reached on around 5 d.p.i., although a few strains were discordant, with maximal production being seen on day 3 (DENV 2DF), 4 (DENV 3DF) or 10 (DENV 3/1113) p.i. In terms of overall growth profile, the profiles for the four DENV 1 (Fig. 1a) and five DENV 4 (Fig. 1d) isolates were remarkably uniform showing similar virus production curves, while the profiles for the two DENV 2 (Fig. 1b) and three DENV 3 (Fig. 1c) isolates were markedly non-uniform, showing different virus production curves. Markedly, the growth profile of the high passage DENV 1 Hawaii strain was similar to the DENV 1 low passage isolates (Fig. 1a). Statistical analysis of the results showed that the actual titers were statistically different from each other for nearly every time point examined, and across all four serotypes (Additional file 2).

**Fig. 2:**
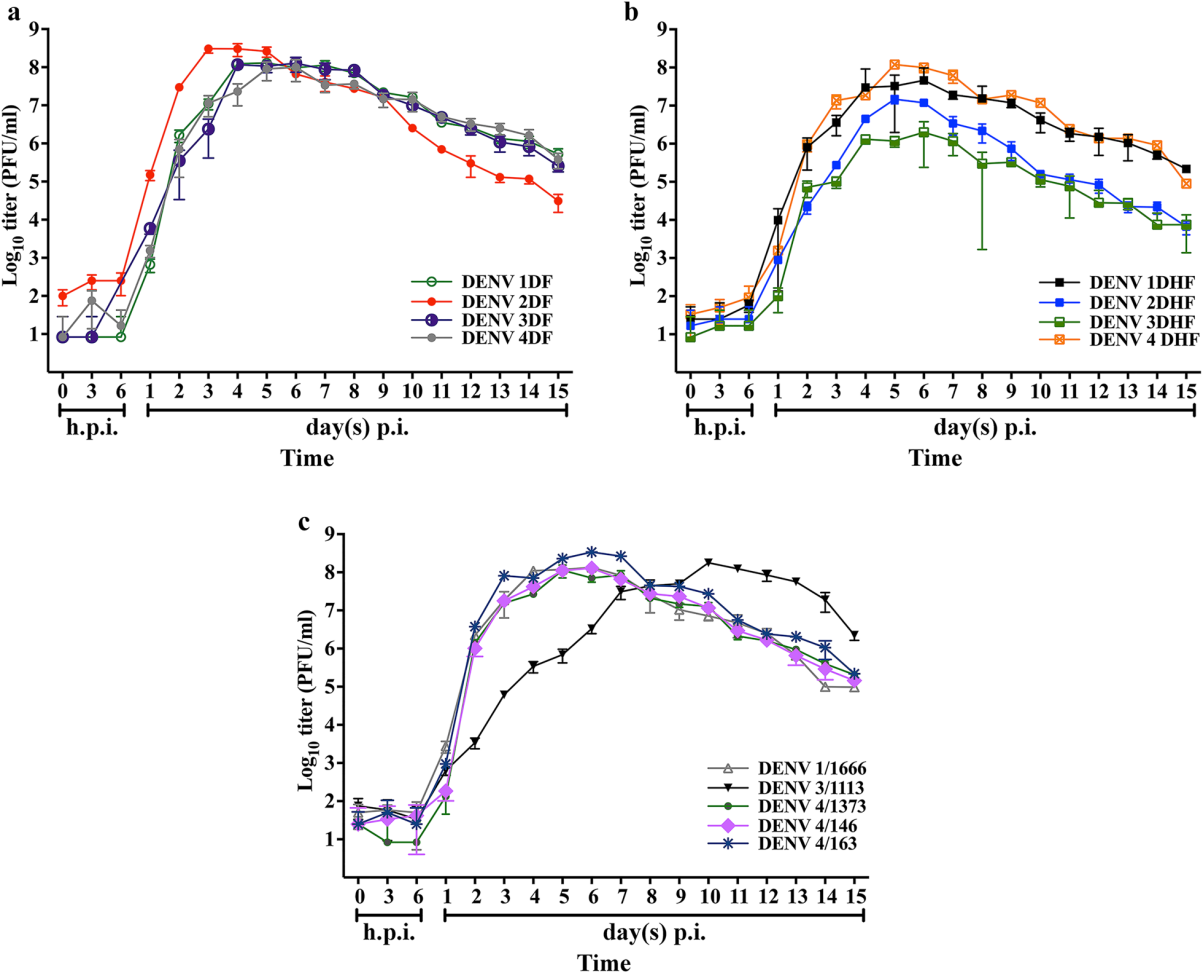
Growth kinetic production profiles of DENV classified by origin. The virus production profiles of different low passage dengue isolates classified by origin. The original sources of DENV included (**a**) four isolates from dengue fever patients (DENV 1DF, DENV 2DF, DENV 3DF, and DENV 4DF) (**b**) four isolates from dengue haemorrhagic fever patients (DENV 1DHF, DENV 2DHF, DENV 3DHF, and DENV 4DHF), and (**c**) five isolates from undifferentiated fever patients (DENV 1/1666, DENV 3/1113, DENV 4/1373, DENV 4/146, and DENV 4/163). The experiment was undertaken independently in triplicate and error bars show mean ± SD. A full statistical analysis of the data is provided in Additional file 2

When analysed by virus origin in terms of disease severity (with the exclusion of the high passage Hawaii strain to its somewhat uncertain provenance [9]) (Fig. 2a–c), the DHF isolates broadly showed a uniform production profile, albeit one that showed the greatest variation in terms of peak titer, which ranged from 6Log10 to 8Log10 which was reached on days 5 and 6 p.i. (Fig. 2b). Maximum titer for the DF isolates was reached on days 3 to 5 p.i., with the DENV 2DF profile showing a 2Log10 increase in virus production over the other isolates between days 1 to 3 p.i. Interestingly, this strain showed the lowest titer of the DF isolates on day 15 p.i. While the isolates from asymptomatic fever patients generally showed a similar virus production profile, the production profile of DENV 3/1113 was markedly different from the other isolates, showing a several Log10 lower titer than other isolates between days 2 and 6 p.i., and showing maximum titer four days later than the other isolates. As with the statistical analysis by serotype, statistical analysis of the results showed that the actual titers were statistically different from each other for nearly every time point examined, and across all severity origins of the viruses (Additional file 2).

While our earlier study on four DENV strains (one for each serotype) undertaken in C6/36 cells showed relatively consistent virus production profiles [4], this study, on a much larger number of isolates shows that there is considerable variation in the replication kinetics of DENV strains. Based on the strains used in this study DENV 1 and DENV 4 strains showed relatively similar virus production profiles, while the results from DENV 2 and DENV 3 isolates show greater variation. However, it cannot be ruled out that other DENV 1 and 4 strains may also show markedly different production profiles. Even less uniformity in production profiles was seen when the analysis was conducted by stratifying by virus origin (based upon disease severity). Overall the results suggest that the variation seen in DENV strains is a significant factor in mediating virus replication, more than serotype or virus origin, supporting our previous observations [5, 6].

### Conclusion

These results support our previous observations that strain variations seen in DENV may significantly impact upon both understanding the mechanism of the virus: host cell interaction, as well as on drug development, and that studies should not underestimate the contribution of DENV variability. However, how the variation affects the virus production profile remains unknown. In particular, care should be taken to not assume that a particular finding with one DENV strain is representative of all DENVs.

## Limitations

This study only looked at 14 viruses, and it is possible that a different picture would emerge if this number was significantly increased. In addition, only one cell line (C6/36) was utilized and this cell line is of an insect origin. A similar study in a mammalian cell line, or indeed in a different insect cell line such as the *Ae*. *aegypti* cell line CCL-125 that we have previously shown to be susceptible to DENV infection [10] might produce different results. Indeed, a previous study looking at four high passage DENVs representative of each serotype showed significantly greater replication variation in the mammalian cell line HepG2 [11] than the same viruses grown in C6/36 cells [4]. Similarly, this study only utilized a single MOI, and the use of both higher and lower MOIs may provide additional insights into DENV replication kinetics.

## Supplementary information

**Additional file 1: Figure S1.** Phylogenetic tree analysis of viruses used in this study. **Table S1.** List of dengue viruses and their provenance.

**Additional file 2:** Sheet 1 Raw data of plaque assay for Fig. 1. Sheet 2 Raw data of plaque assay for Fig. 2. Sheet 3 Statistical analysis of Fig. 1 data. Sheet 4 Statistical analysis of Fig. 2 data.

## Data Availability

All data generated or analysed during this study are included in the published article and its supplementary information file.

## Abbreviations

*Ae*: *Aedes*
DF: Dengue fever
DHF: Dengue haemorrhagic fever
DSS: Dengue shock syndrome
DENV: Dengue virus
