# Supplementary material for: Analysis of the virus propagation profile of 14 dengue virus isolates in Aedes albopictus C6/36 cells
Source: BMC Res Notes. 2020 Oct 12;13:481. doi: 10.1186/s13104-020-05325-6 (PMC7552352; doi:10.1186/s13104-020-05325-6)
Supplement: Supplementary file 1 — Additional file 1: Figure S1. Phylogenetic tree analysis of viruses used in this study. Table S1. List of dengue viruses and their provenance. [file 13104_2020_5325_MOESM1_ESM.pdf]

Additional file 1

Analysis of the virus propagation profile of 14 dengue virus isolates in *Aedes albopictus* C6/36 cells.

Atitaya Hitakarun[1], Suwipa Ramphan[1], Nitwara Wikan[1], and Duncan R. Smith[1]*

[1]Institute of Molecular Biosciences, Mahidol University, Bangkok, 73170, Thailand

*Correspondence to Duncan R. Smith, Molecular Pathology Laboratory, Institute of Molecular Biosciences, Mahidol University, Salaya Campus, 25/25 Phuttamonthon Sai 4, Salaya, Nakhon Pathom, Thailand 73170; Phone: 66(0) 2441-9003 to 7. Fax: 66 (0) 2441-1013. E-mail: duncan_r_smith@hotmail.com, duncan.smi@mahidol.ac.th

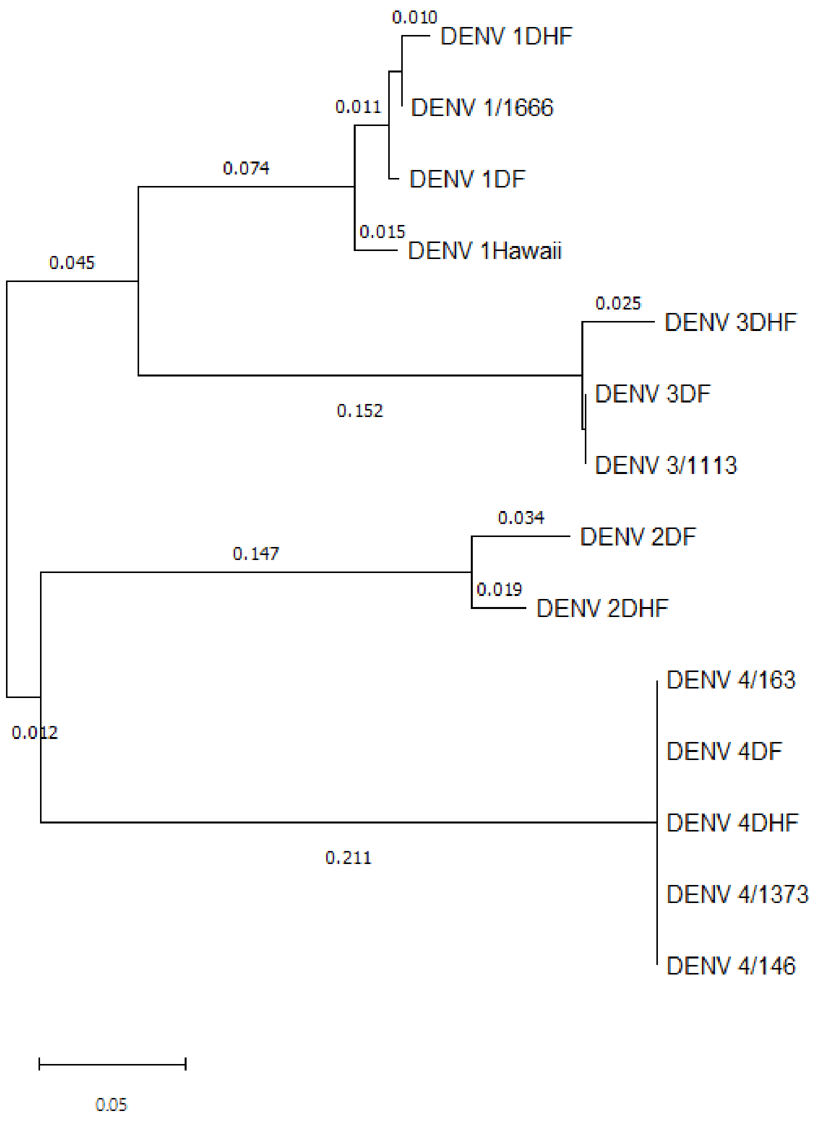

Supplemental Figure 1. A phylogenetic tree generated using partial sequences of viruses used in this study. The tree was generated using the MEGA program version 10.1.6 and the Maximum Likelihood method.

Supplemental Table 1. List of dengue viruses and their provenance

Serotype	Strain	Designation	Year Isolated	Clinical grading	Passage history
DENV 1	Hawaii	DENV 1Hawaii	1944	-	High passage
	SS12/61	DENV 1DF	2006	DF	Low Passage
	SS12/60	DENV 1DHF	2006	DHF	Low Passage
	SS11/1666	DENV 1/1666	2010	Undifferentiated fever	Low Passage
DENV 2	SS12/62	DENV 2DF	2006	DF	Low Passage
	SS12/63	DENV 2DHF	2006	DHF	Low Passage
DENV 3	SS12/64	DENV 3DF	2006	DF	Low Passage
	SS12/65	DENV 3DHF	2006	DHF	Low Passage
	SS15/1113	DENV 3/1113	2015	Undifferentiated fever	Low Passage
DENV 4	SS12/66	DENV 4DF	2006	DF	Low Passage
	SS12/67	DENV 4DHF	2006	DHF	Low Passage
	SS11/1373	DENV 4/1373	2009	Undifferentiated fever	Low Passage
	SS14/146	DENV 4/146	2013	Undifferentiated fever	Low Passage
	SS14/163	DENV 4/163	2014	Undifferentiated fever	Low Passage

"-" = symptoms compatible with dengue (originally from pooled blood of six people with symptoms compatible with dengue). All viruses except DENV 1 Hawaii were isolated in Thailand.
